# Consequences of polar form coherence for fMRI responses in human visual cortex

**DOI:** 10.1016/j.neuroimage.2013.04.036

**Published:** 2013-04-19

**Authors:** Damien J. Mannion, Daniel J. Kersten, Cheryl A. Olman

**Affiliations:** a Department of Psychology, University of Minnesota, Minneapolis, MN, USA; b Department of Brain and Cognitive Engineering, Korea University, Seoul, Republic of Korea

**Keywords:** fMRI, Visual cortex, Orientation, Spatial vision

## Abstract

Relevant features in the visual image are often spatially extensive and have complex orientation structure. Our perceptual sensitivity to such spatial form is demonstrated by polar Glass patterns, in which an array of randomly-positioned dot pairs that are each aligned with a particular polar displacement (rotation, for example) yield a salient impression of spatial structure. Such patterns are typically considered to be processed in two main stages: local spatial filtering in low-level visual cortex followed by spatial pooling and complex form selectivity in mid-level visual cortex. However, it remains unclear both whether reciprocal interactions within the cortical hierarchy are involved in polar Glass pattern processing and which mid-level areas identify and communicate polar Glass pattern structure. Here, we used functional magnetic resonance imaging (fMRI) at 7T to infer the magnitude of neural response within human low-level and mid-level visual cortex to polar Glass patterns of varying coherence (proportion of signal elements). The activity within low-level visual areas V1 and V2 was not significantly modulated by polar Glass pattern coherence, while the low-level area V3, dorsal and ventral mid-level areas, and the human MT complex each showed a positive linear coherence response functions. The cortical processing of polar Glass patterns thus appears to involve primarily feedforward communication of local signals from V1 and V2, with initial polar form selectivity reached in V3 and distributed to multiple pathways in mid-level visual cortex.

## Introduction

Units in low-level regions of the cortical visual hierarchy each receive stimulation from a restricted portion of the visual field. Yet important features in the visual image are often spatially extensive and have complex (non-Cartesian) form structure that is not necessarily apparent in such local representations. Glass (1969) provided a compelling demonstration of our sensitivity to such complex spatial structure in his eponymous patterns; if a dense field of randomly-positioned dots is combined with a duplicate that has been displaced at a polar angle (rotated, for example), we can perceive spatial form consistent with the geometric transformation (see Fig. 2 for examples). This perception arises despite the representation of the polar structure in the local responses being noisy and ambiguous (due to each dot having many potential corresponding partner dots), which implies the existence of cortical pathways that spatially aggregate local responses and are attuned to complex spatial form.

The perception of polar form in Glass patterns has been conceived as arising from the feedforward transmission of locally filtered signals to a spatial pooling and complex-form selective mechanism (Wilson and Wilkinson, 1998; Wilson et al., 1997). However, recent neuroimaging evidence indicates that stimuli with similar requirements for multiple processing stages may involve reciprocal interactions rather than pure feedforward communication. In particular, increased activity within cortical areas higher in the visual hierarchy can be accompanied by reduced activity in low-level areas containing the local representations (Cardin et al., 2011; Fang et al., 2008; Murray et al., 2002). Although the putative functional role of such interactions, to reduce ambiguity in the local representations and to enhance the efficiency of cortical activity (Murray et al., 2004), appear applicable and desirable for polar Glass pattern processing circuitry, it is uncertain whether low-level activity is indeed affected by the presence of complex spatial structure in polar Glass patterns.

The visual areas that identify and communicate the presence of complex form in polar Glass patterns are also unclear. The multistage model of polar Glass pattern processing nominates ventral visual cortex as the presumptive site of spatial pooling and complex form selectivity (Wilson and Wilkinson, 1998; Wilson et al., 1997). Consistent with this proposal, neuroimaging evidence shows the mid-level ventral area human V4 (hV4) to be selective for particular polar spatial forms (Wilkinson et al., 2000), including when instantiated in Glass patterns (Mannion and Clifford, 2011; Mannion et al., 2010; Ostwald et al., 2008). Furthermore, a patient with a lesion near the hV4 region of the ventral stream has greatly diminished capacity for perceptually discriminating polar from random Glass patterns (Gallant et al., 2000). However, sensitivity to polar Glass patterns has also been reported in mid-level dorsal areas (Mannion and Clifford, 2011; Ostwald et al., 2008; Swettenhamet al., 2010). The contribution of such dorsal stream areas to the complex form in polar Glass patterns remains unknown.

Here, we aimed to reveal the network of brain areas in low-level and mid-level visual cortex responsive to polar Glass patterns and to infer the interactions within the cortical hierarchy that support such selectivity. We used functional magnetic resonance imaging (fMRI) at 7 T to measure the blood oxygen level dependent (BOLD) activity in human visual cortex while participants viewed polar Glass patterns of varying coherence. At zero coherence, each dot pair is oriented at a random polar angle and hence no perception of coherent complex form is elicited. Increasing coherence involves a greater proportion of dot pairs following a particular polar transformation, with a corresponding increase in the strength of the complex spatial form signals. This parametric manipulation supplies a rich characterization of sensory responses in human visual cortex, as has previously been obtained with motion (Rees et al., 2000), contour (Dumoulin and Hess, 2006), and stereo (Chandrasekaran et al., 2007) stimuli, to inform our understanding of polar Glass pattern processing.

## Material and methods

### Participants

Six observers (four female), each with normal or corrected-to-normal vision, participated in the current study. Each participant gave their informed written consent and the study conformed to safety guidelines for MRI research and was approved by the Institutional Review Board at the University of Minnesota.

### Apparatus

**Fig. 1:**
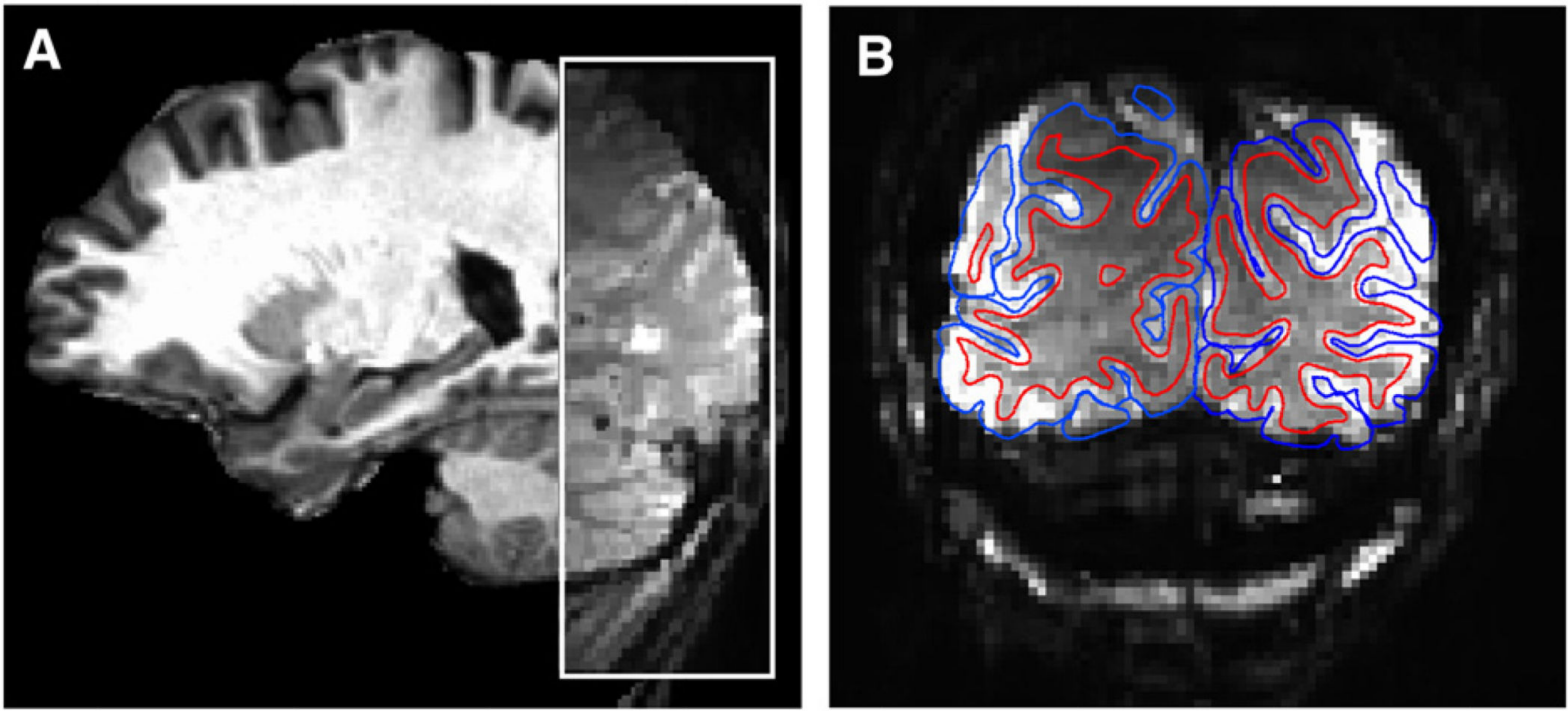
Example functional image acquisitions. (A) Sagittal view of an example session-mean (pre-processed) functional image overlaid on the associated anatomical image. (B) Coronal (inplane) view of a session-mean functional image, showing white matter (red) and pial (blue) surfaces obtained from segmentation of a coregistered anatomical image.

Functional imaging was conducted using a 7T magnet (Magnex Scientific, UK) with a Siemens (Erlangen, Germany) console and head gradient set (Avanto). Images were collected with a $T_{2}^{*}$ sensitive gradient echo imaging pulse sequence (TR = 2 s, TE = 18 ms, flip angle = 70°, matrix = 108 × 108, GRAPPA acceleration factor = 2, FOV = 162 × 162 mm, partial Fourier = ⅞, voxel size = 1.5 mm isotropic) in 36 ascending interleaved coronal slices covering the occipital lobes (see Fig. 1).

Stimuli were displayed on a screen positioned within the scanner bore using a VPL-PX10 projector (Sony, Tokyo, Japan) with a spatial resolution of 104 × 768 pixels, temporal resolution of 60 Hz, and mean luminance of 168 cd/m^2^. Participants viewed the screen from a distance of 72 cm, via a mirror mounted on the head coil, giving a viewing angle of 29.1° × 21.8° that accommodated a visible square region of approximately 14.5° diameter due to occlusion from the scanner bore. Stimuli were presented using PsychoPy 1.73.05 (Peirce, 2007). Behavioral responses were indicated via a FIU-005 fiber optic response device (Current Designs, PA). As detailed below, analyses were performed using FreeSurfer 5.1.0 (Dale et al., 1999; Fischl et al., 1999), FSL 4.1.6 (Smith et al., 2004), and AFNI/SUMA (2012/11/23; Cox, 1996; Saad et al., 2004). Experiment and analysis code is available at http://bitbucket.org/djmannion/glass_coherence_block.

### Stimuli

**Fig. 2:**
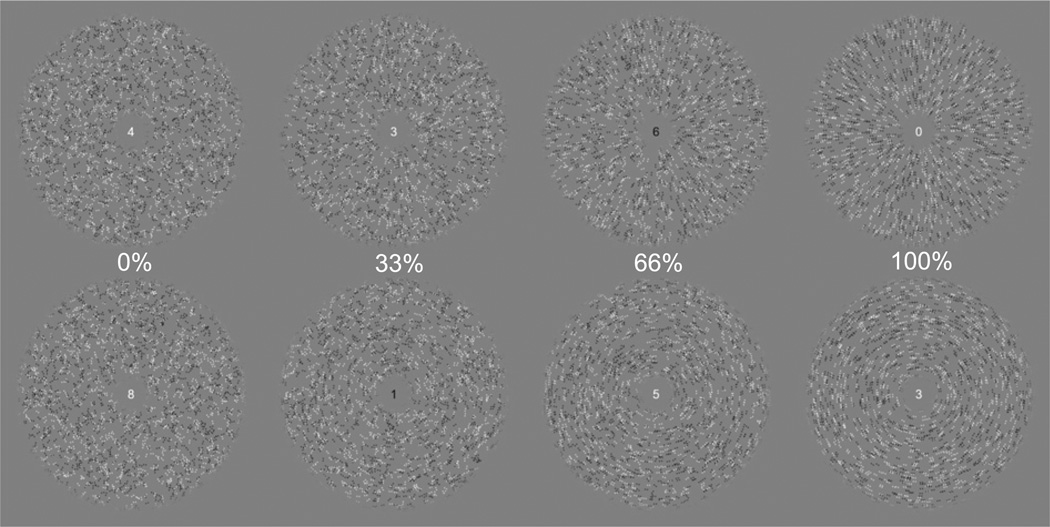
Stimulus conditions and display layout. Glass patterns were shown in an annular aperture and were constructed to give a perception of starburst (top row) or circular (bottom row) polar form, with the strength of the polar form percept moderated by the pattern coherence (columns; 0%, 33%, 66%, or 100% of the dot pairs aligned with the polar form, the remainder were oriented randomly). Digits relating to a behavioral task were presented at central fixation.

Each stimulus consisted of a Glass pattern within an annular aperture surrounding central fixation, as shown in Fig. 2. Each Glass pattern was constructed by assigning random positions to a set of dipoles (dot pairs) based on a uniform allocation over image area with a dot density of 25 dots/° visual angle^2^. Each dipole was designated as either signal or noise such that the total matched the desired level of pattern coherence; for example, a pattern of 33% coherence comprised 33% signal dipoles. Dipole elements (dots) were placed 0.14° visual angle apart at an orientation consistent with the desired polar form for signal dipoles (either circular or starburst, see Design) and at a random angle for noise dipoles. Each dot had a Gaussian profile (σ = 0.025° visual angle), and each dipole was randomly assigned to have its peaks be either a full contrast increment or decrement from the mean-luminance background. Glass patterns were presented within an annular aperture with an outer diameter of 14.4° visual angle, an inner diameter of 1.5° visual angle, and with reduced contrast in the 0.75° visual angle before the inner and outer edges following a raised cosine profile. While this stimulus extent is larger than necessary to perceive coherent polar form, spatial integration can proceed over considerable regions of the visual field (Dickinson et al., 2009) and fMRI response estimation reliability for a given visual area is enhanced by the capacity to measure from units with receptive field locations tiling the visual field. Digits relating to a concomitant behavioral task were drawn in the center of the stimulus annulus.

### Design

The experiment followed a block design protocol with four stimulus conditions, pattern coherences of 0%, 33%, 66%, and 100% (see Fig. 2 for examples of different coherence levels), and a baseline condition in which no pattern was displayed. Within each stimulus block, a new Glass pattern instantiation was presented at 1 Hz with a 750 ms on/250 ms off visibility profile. The signal dipole orientation was randomly assigned for each instantiation to be consistent with either circular or starburst form (see Fig. 2 for examples of circular and starburst patterns). Both circular and starburst patterns were included to limit potential adaptation effects on the coherence response, as repetitive viewing of Glass patterns of consistent form biases perception of subsequent patterns (including those with no coherent structure) towards a pattern of orthogonal orientation (Clifford and Weston, 2005), and to balance their orientation structure relative to fixation (Mannion and Clifford, 2011; Mannion et al., 2010). Each block was 16 s in duration, and blocks were ordered in sequences in which the four stimulus condition blocks were followed by a blank block, with the arrangement of stimulus blocks chosen such that each condition was preceded an equal number of times by each of the other conditions (Clifford et al., 2009). There were four such sequences per run, and an additional 6 s blank period was prepended and a blank block was appended to the run order, giving a run duration of 342 s (171 volumes). Each participant completed 12 runs, collected within a single session.

Participants engaged in a challenging behavioral task during each run. A digit, randomly chosen from the set of zero to nine and of random polarity (black or white), was presented at fixation (see Fig. 2) and updated at 3 Hz (Goddard et al., 2010). Participants were required to respond, with a button press, when the digit matched a known target in both digit and polarity. Two targets, of opposite polarities and differing digits, were chosen randomly at the beginning of each functional run and displayed to the participant. This task had no direct relevance to the aims of this study, but was designed to divert participant's attention away from the stimuli and hence to limit the effects of potentially unequal attentional allocation to differing pattern coherences.

### Anatomical acquisition and processing

A T_1_-weighted anatomical image (sagittal MP-RAGE, 1 mm isotropic resolution) was collected from each participant in a separate session using a Siemens Trio 3T magnet (Erlangen, Germany). FreeSurfer (Dale et al., 1999; Fischl et al., 1999) was used for segmentation, cortical surface reconstruction, and surface inflation and flattening of each participant's anatomical image.

### Visual area definition

**Fig. 3:**
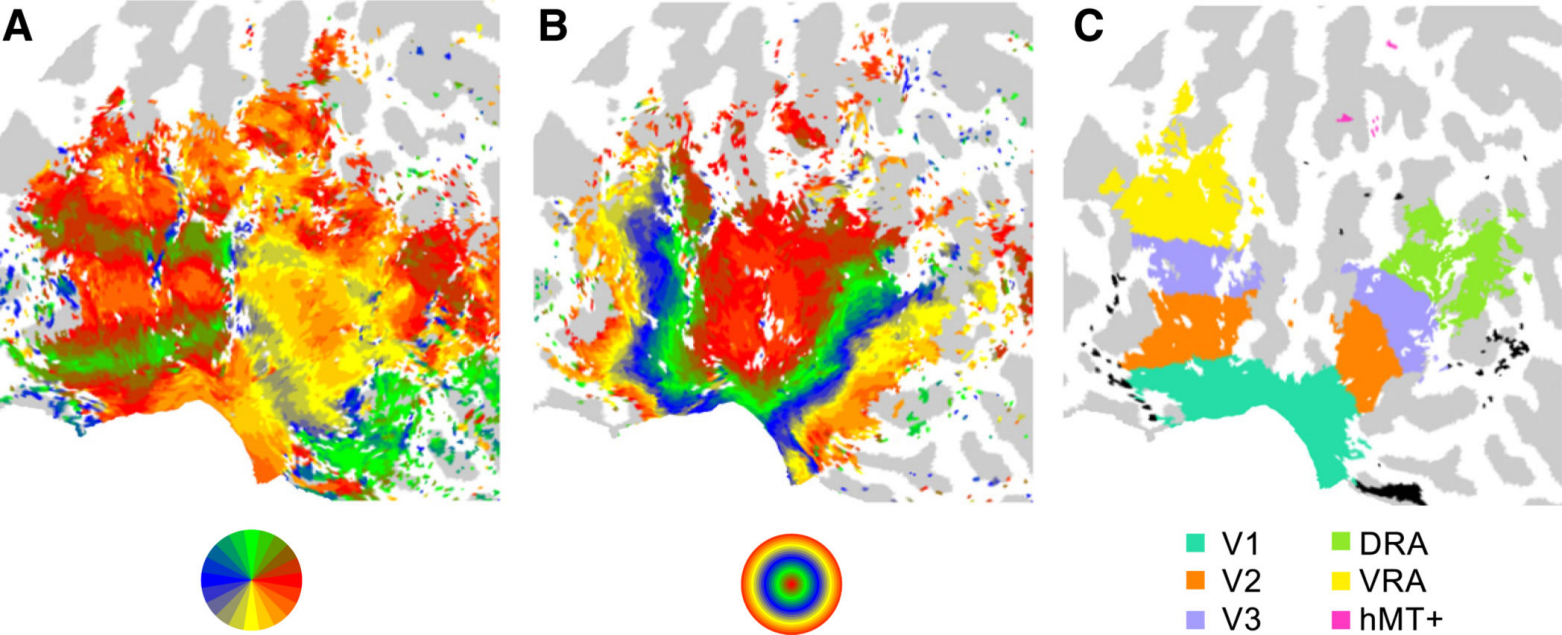
Parcellation of human visual cortex. (A) Angular visual field preference obtained from rotating wedge stimulation. (B) Eccentricity visual field preference obtained from expanding/contracting ring stimulation. (C) Regions significantly responsive to Glass pattern stimulation, colored according to their associated visual area (unassigned activation shown in black). All panels show a flattened representation of an example participant's posterior left hemisphere.

Visual areas were defined based on analysis of functional acquisitions, obtained in a separate scanning session, that followed standard procedures for the parcellation of human visual cortex. Participants observed four runs of a clockwise/anti-clockwise rotating wedge stimulus and two runs of an expanding/contracting ring stimulus (DeYoe et al., 1996; Engel et al., 1997; Hansen et al., 2007; Larsson and Heeger, 2006; Schira et al., 2007; Sereno et al., 1995), and the data was analyzed via phase-encoding methods (Engel, 2012) to establish visual field preferences over the cortical surface (as shown for an example participant in Fig. 3). The angular and eccentricity phase maps were used to manually define the low-level visual areas V1, V2, and V3.

An additional two runs contrasted blocks of translating and static low-contrast dots to identify the human MT complex (Tootell et al., 1995). Furthermore, two runs in which the stimulus alternated between intact and scrambled objects (Larsson and Heeger, 2006) were consulted to interpret observed activation with reference to the lateral occipital complex (LOC; Malach et al., 1995).

### Pre-processing

Functional images were motion corrected using AFNI, with reference to the volume acquired closest in time to a within-session fieldmap image, and resampled with heptic interpolation before being unwarped using FSL to correct geometric distortions introduced by magnetic field inhomogeneities. The participant's anatomical image was then coregistered with a mean of all functional images via AFNI's align_epi_anat.py, using a local Pearson correlation cost function (Saad et al., 2009) and six free parameters (three translation, three rotation). Coarse registration parameters were determined manually and passed to the registration routine to provide initial estimates and to constrain the range of reasonable transformation parameter values. The motion-corrected and unwarped functional data were then projected onto the cortical surface by averaging between the white matter and pial boundaries (identified with FreeSurfer) using AFNI/SUMA. All analysis was performed on the nodes of this surface domain representation.

### Analysis

Functional image analysis was conducted within a general linear model (GLM) framework using AFNI. Stimulus blocks for each condition were modeled as boxcars and convolved with SPM's canonical hemodynamic response function. Legendre polynomials up to the third degree and participant movement estimates were included as additional regressors. The first three volumes (6 s) of each run were censored in the analysis, leaving 2016 data timepoints (168 per run for 12 runs) and 58 regressors (4 conditions, 48 polynomials, and 6 head motion regressors) in the design matrix. The GLM was estimated via AFNI's 3dREMLfit, which accounts for noise temporal correlations via a voxelwise ARMA(1,1) model.

The stimulus condition beta weights obtained from the GLM were converted to percent signal change (psc) via division by the average of the Legendre polynomial regressor timecourse. For each visual area, such percent signal change values were then averaged across the nodes within the area that showed above-baseline responses to visual stimulation (identified by an all stimulus >0 contrast, *p* < 0.001 [FDR corrected, calculated separately across hemispheres]; see Fig. 3). Values within each area were then normalized by subtracting each participant's mean response across stimulus conditions.

The effect of Glass pattern coherence on the BOLD response in each area was evaluated via planned contrasts using linear, quadratic, and cubic orthogonal polynomials. The statistical significance of the resulting trend coefficients was estimated by permutation testing. For each of 10^4^ iterations, the relationship between stimulus condition and measured response was shuffled for each participant and the relevant contrast coefficient computed. The resulting distribution was used to assign a probability of chance alone producing the observed contrast coefficient. Such probabilities were doubled (two-tailed) to encompass sensitivity to trends in both directions. A similar procedure was used to analyze the effect of distance from the vertical visual field meridian on the linear contrast coefficient of V3 nodes (see Results).

We quantified participant performance on the behavioral fixation task by correlating target events with participant responses. The duration of each run was discretized in 100 ms bins and target presence, participant response, and stimulus condition were recorded for each bin. After concatenating across runs, Pearson correlation coefficients were calculated between target and response for bins corresponding to each of the stimulus conditions and for response lags of between 0 and 1.5 s. A two-way repeated measures ANOVA was conducted on the correlations, with stimulus condition (4 levels) and response lag (15 levels) as fixed factors and participants as a random factor.

## Results

We first identified regions of low and mid-level visual cortex that were responsive to visual stimulation within the stimulus annulus. For each participant, we identified nodes (positions on the cortical surface) with a significantly positive (*p* < 0.001, hemisphere FDR corrected) coefficient in a contrast comparing the sum of the response to pattern stimuli against the response to a mean-luminance field. We note that this comparison indexes responsiveness to the current stimulus composition and geometry—it does not carry any implication of selectivity for Glass patterns relative to other forms of stimulation. The resulting maps of activation were consistent across participants, and a representative example is shown in Fig. 3. A band of activation was elicited across the low-level retinotopic areas (V1, V2, and V3), consistent with the annular stimulus geometry. Activation extended beyond V3 into mid-level visual areas both dorsally and ventrally; dorsal activation appeared consistent with foci in visual areas V3A/B and LO1/2 (Larsson and Heeger, 2006) and ventral activation appeared to lie within hV4 (Goddard et al., 2010; Wade et al., 2002) and VO1/2 (Arcaro et al., 2009). We grouped such mid-level activation into dorsal and ventral responsive areas (DRA and VRA) based on their anatomical location, as the nodes could not be confidently assigned to their constituent visual area in all participants. Activation was also reliably present in hMT+, while the LOC was infrequently activated and was not considered further.

**Fig. 4:**
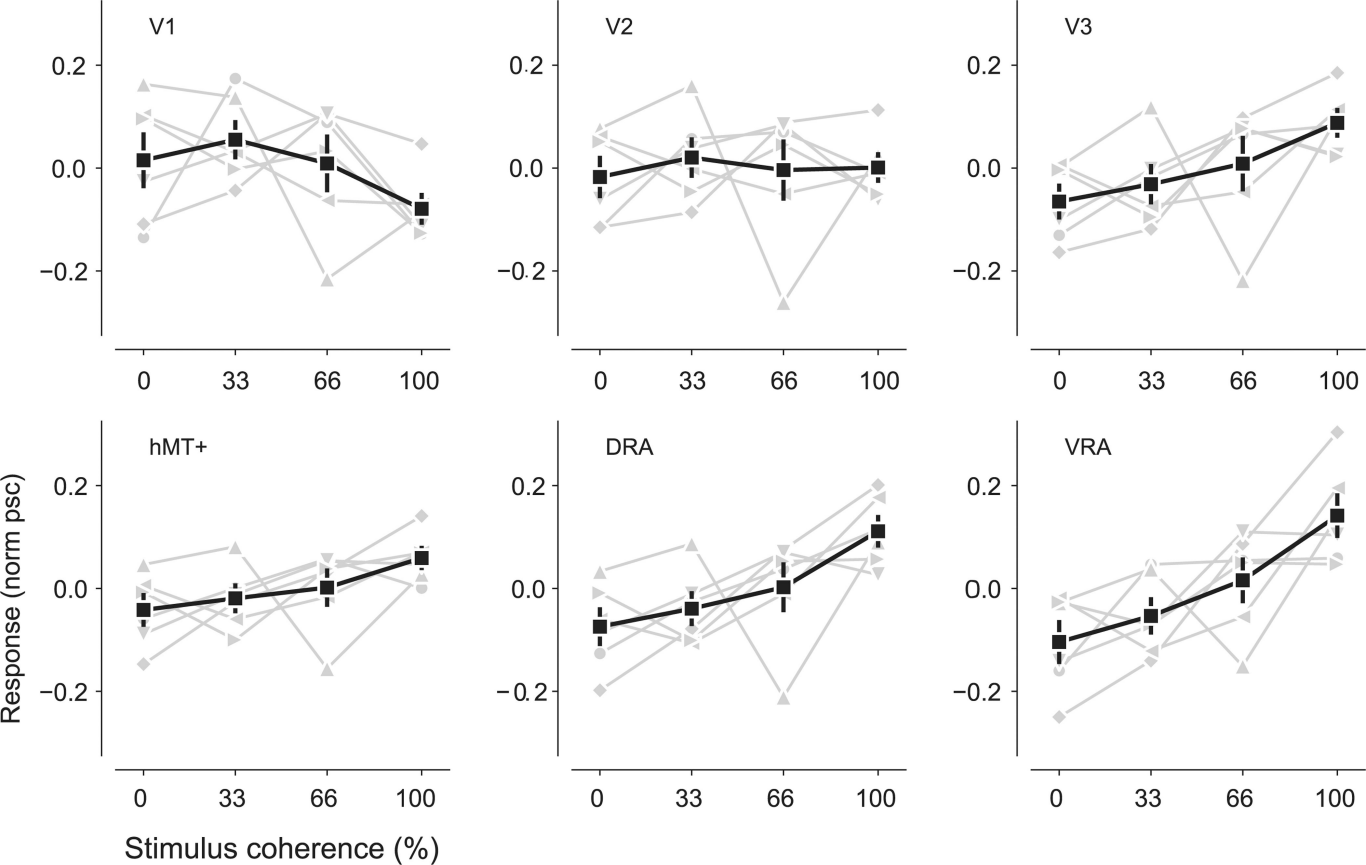
Visual area coherence response functions. Black lines and squares show the mean response (±SEM), across participants, at each stimulus coherence. Gray lines show the response of each participant, with each participant identified by a consistent symbol across all panels. Responses are in units of percent signal change from mean-luminance baseline, normalized to the participant mean across coherences.

To examine the effect of polar form coherence on activity magnitude, we averaged the response (percent signal change, relative to mean-luminance field) within activated nodes of each area to each pattern coherence. Such coherence response functions, after normalization to each participant's mean across coherences, are shown in Fig. 4. We quantified the shape of the coherence response functions by calculating linear, quadratic, and cubic trend coefficients, as described in Table 1. Among the low-level visual areas, only V3 showed a significant effect of coherence, via a positive linear trend (*p* = 0.009) in which full coherence elicited an average of 0.153 psc (SEM = 0.046) higher response than zero coherence. Both the mid-level areas, DRA and VRA, showed significant effects of coherence evident in positive linear trends (DRA: *p* = 0.003, VRA: *p* < 0.001), with an average of 0.185 psc (SEM = 0.054) and 0.246 psc (SEM = 0.067) higher response for full coherence relative to zero coherence for DRA and VRA, respectively. A significant positive linear trend was also present in hMT + (*p* = 0.025), with an average of 0.101 psc (SEM = 0.042) higher response to full coherence relative to zero coherence. No area under consideration showed a significant quadratic or cubic trend (all *p* ≫ 0.05).

As visual area V3 borders DRA and VRA, we were concerned that its apparent linear increase in response with increasing polar form coherence could reflect misattribution of mid-level area responses, which neighbor V3 on the cortical surface, rather than an intrinsic V3 property. If present, such a confound should be limited to nodes with a retinotopic preference close to the vertical meridian in the visual field, as this area is shared with DRA (lower visual field) and VRA (upper visual field) while the horizontal meridian is shared with V2 (see Fig. 3). The linear contrast coefficient remained significant when considering V3 nodes representing the 0 to 22.5° (*p* = 0.003), 22.5 to 45° (*p* = 0.009), and 45 to 67.5° (*p* = 0.010) angular distance from the vertical meridian. The linear contrast coefficient was less reliable (*p* = 0.054) for V3 nodes 67.5 to 90° circular distance from the vertical meridian. However, the maintenance of significant linear trends for V3 subdivisions distinct from the border with mid-level areas makes it unlikely that the observed V3 responses only reflect the profiles of neighboring mid-level areas.

**Fig. 5:**
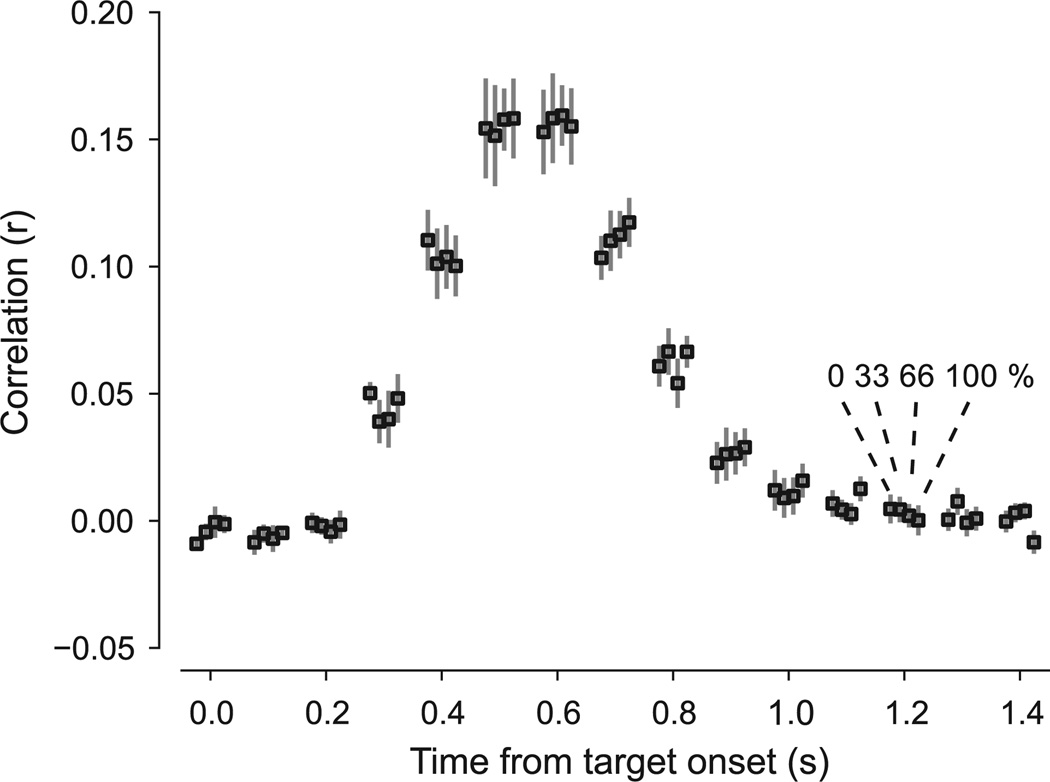
Performance on behavioral task at fixation. Performance was quantified as the correlation between target presence and participant response (vertical axis) at different time lags (horizontal axis). At each time lag, performance is shown as the mean ± SEM across participants for targets occurring during presentation of the different stimulus coherence levels (displaced horizontally about each time lag).

We also wanted to ensure that any significant trends in the observed coherence response functions reflected sensory processing rather than unequal attentional allocation. We used performance on the fixation task as a measure of attentional engagement, quantified by the correlation between target presence and observer response. As shown in Fig. 5, performance was highest at a response lag of around 500 ms and was comparable across stimulus conditions. A two-way within-subjects ANOVA indicated a significant main effect of response lag (*F*_14,280_ = 181.59, *p* < 0.001), while neither the main effect of stimulus condition or the interaction between stimulus condition and response lag were statistically significant (both *p* ≫ 0.05). This is consistent with participants being engaged with the task and performing similarly during different stimulus conditions, hence rendering confounding effects of attention unlikely.

## Discussion

We investigated how observation of polar Glass patterns of varying coherence affects the magnitude of the fMRI BOLD signal in low(V1, V2, and V3) and mid level (dorsal, ventral, and hMT+) human visual cortex. We report that V1 and V2 were not notably affected by Glass pattern coherence, while the low-level region V3 and each of the evaluated mid-level regions (DRA, VRA, hMT+) displayed a positive linear increase in response with increasing coherence from random to fully coherent polar Glass patterns.

Our first aim in conducting this study was to investigate whether low-level visual areas are affected by the extraction of complex spatial structure in higher areas of the cortical hierarchy, as evident in other stimulus paradigms where processing is distributed across different visual areas (Cardin et al., 2011; Fang et al., 2008; Murray et al., 2002). The lack of notable deviation from a flat coherence response function observed in V1 and V2 is consistent with the feedforward communication of local signals from low-level visual areas without any strong influence of coherence modulations observed in mid-level visual cortex. Perhaps this discrepancy can be reconciled by considering the potentially limited utility of local information in polar Glass patterns; if an important functional role of feedback processing is to disambiguate local representations once a higher-level interpretation has been reached (Epshtein et al., 2008), the spatially random positioning of local signals may not warrant the engagement of feedback circuitry. Alternatively, if feedback reduces the low-level response to stimulus aspects captured by higher level areas (‘explaining away’; Murray et al., 2004), rather than sharpening the response by suppressing inconsistent signals, the low proportion of local signals consistent with the polar Glass pattern form may render such modulations difficult to detect in the pooled fMRI response.

The apparent lack of deviation from flat coherence response functions in V1 and V2 observed here is consistent with electrophysiological recordings from macaque V1 (Smith et al., 2002) and V2 (Smith et al., 2007). However, it appears inconsistent with previous reports of differential responses to the type of polar form (circular through spiral to starburst) in these areas (Mannion and Clifford, 2011; Mannion et al., 2010). With comparable stimulus geometry, Mannion et al. (2010) showed that V1 and V2 responses are highest to circular and starburst polar Glass patterns and lowest to spirals. This polar form selectivity could potentially be reconciled with the flat coherence response functions observed in the current study, however it would require a reduced response to spiral compared to random Glass patterns in V1 and V2 that has not been evaluated. Alternatively, the continuous presentation paradigm used by Mannion et al. (2010) may have rendered the responses particularly sensitive to the subpopulation of low-level units that possess polar form tuning (Hegdé and Van Essen, 2007; Mahon and De Valois, 2001).

The visual area V3 was the first area in the presumptive visual hierarchy to show selectivity for polar Glass patterns, with a positive increasing response to increasing coherence. This selectivity is consistent with Mannion and Clifford (2011), who found that V3's anisotropic response to different polar Glass pattern forms was more similar to that of mid-level regions than V1 and V2, and with reports that random-appearing and polar Glass patterns that are dynamic (rapidly updated sequence of new Glass pattern instances) are first differentiated in V3 (Krekelberg et al., 2005). We do not consider the observed V3 coherence selectivity to simply reflect an increase in receptive field sizes with ascension of the visual hierarchy. The difference in the distributions of receptive field sizes that contribute to the average response of V1, V2, and V3 is likely to be small, as the increase in receptive field size with eccentricity within low-level visual areas (Smith et al., 2001) causes the majority of receptive field sizes in V3 to also be present (in more peripheral locations) in V1 and V2. Instead, the significant coherence response in V3 may have its substrate in V3's positioning to receive input from the local orientation extraction performed in V1 and V2 in combination with the complex orientation selectivity exhibited by V3 subpopulations reported by Felleman and Van Essen (1987). Although the organization and functional properties of V3 remain controversial (Lyon and Kaas, 2002; Wandell et al., 2007; Zeki, 2003), the V3 region of human visual cortex may play a more profound role in the processing of polar Glass patterns than simple relaying of local information from low-level to mid-level areas.

The second aim of the current study was to evaluate the responses of mid-level areas of the visual hierarchy to polar Glass patterns. The strong modulation by polar Glass pattern coherence in ventral midlevel areas (VRA) agrees with previous reports of sensitivity to such forms in area hV4 (Mannion and Clifford, 2011; Mannion et al., 2010; Wilkinson et al., 2000) and supports the proposed role of the ventral stream in the multistage model of Glass pattern perception (Wilson and Wilkinson, 1998; Wilson et al., 1997). Furthermore, the apparent positive linearity of the observed VRA coherence response agrees with the predictions of ventral stream responses in the neural model described by Wilson and Wilkinson (1998).

However, we also find a strong positive linear coherence response in mid-level dorsal areas (DRA). This DRA selectivity is consistent with previous reports of polar form anisotropies in V3A/B (Mannion and Clifford, 2011) and a preference for polar form Glass patterns over random in the region of V3A/B with fMRI (Ostwald et al., 2008) and magnetoencephalography (Swettenham et al., 2010). We also find a positive linear coherence response in the human MT complex, consistent with reported polar form selectivity in this area for dynamic Glass patterns (Krekelberg et al., 2005). As dynamic Glass patterns yield an impression of motion (Ross et al., 2000), the comparatively slow update rate of the presentation in the current study—which produced no motion percept—extends the response properties of hMT + to encompass polar form independent of perceived motion.

Thus, polar Glass pattern coherence has consequences for activity in multiple areas of human mid-level visual cortex. We speculate that this processing in multiple pathways may reflect different perspectives on the Glass pattern structure. For areas in the ventral stream, the organization of paired dots may arise from the reflectance structure, or texture, of a surface, and identification of this organization would be informative regarding the surface's material composition and structural arrangement. Given the disruptive influence of a ventral stream lesion on polar Glass pattern perception (Gallant et al., 2000), this is likely to be the pathway underlying our conscious impression of spatial form while observing such patterns. In contrast, dorsal areas and hMT + may consider polar Glass patterns as resembling a field of elements with a flowing structure induced by either self-motion (forward/backward or rotational) or due to the influence of external forces. This view is consistent with the description of Glass patterns as ‘static flow’ (Kovaćs and Julesz, 1992), and that a perception of motion is obtained with dynamic Glass patterns (Krekelberg et al., 2005; Ross et al., 2000). Perhaps the polar form could be recovered from such Glass patterns despite lesioned ventral areas (Gallant et al., 2000), or learned by ordinary observers under conditions of reduced visibility (Roseboom and Arnold, 2011), if task demands required an active response such as reproducing the transformation with head movement.

This presence of polar Glass pattern modulations in multiple mid-level areas suggests a potentially pivotal role for V3. Extracting the key aspects of scene analysis in V3 avoids duplicating common processing in divergent higher-level areas, allowing them to instead concentrate on specialized analyses relevant to their pathway. In monkeys, V3 projects to the ventral area V4, the dorsal area V3A, and to MT (Felleman et al., 1997), and thus appears to possess the requisite circuitry for such a role. This view of V3 as a hub for information being routed to multiple higher-level areas has also recently been proposed by Lescroart and Biederman (2013) regarding object perception, and future research is required to elaborate on this intriguing possibility.

In summary, we have detailed the responses of low and mid level human visual cortex to stimulation with polar Glass patterns of increasing coherence. We did not find strong evidence for polar form selective responses in V1 and V2, consistent with the feedforward communication of noisy and ambiguous local information from these areas. The responses of V3, mid-level dorsal and ventral, and hMT + each show a positively increasing coherence response. This finding elaborates the cortical network involved in processing polar Glass patterns beyond the ventral stream, and suggests a pivotal role for V3 in communicating complex image structure to higher-level areas of the brain.

## Figures and Tables

**Table 1:** Linear, quadratic, and cubic coefficients and probability values for coherence response functions from each visual area under consideration.

Area	Linear		Quadratic		Cubic	
	[−3,−1,+1,+3]		[+1,−1,−1,+1]		[−1,+3,−3,+1]	
	Coeff.	*p*	Coeff.	*p*	Coeff.	*p*
V1	− 0.329	0.145	− 0.128	0.204	0.044	0.840
V2	0.031	0.883	− 0.033	0.715	0.091	0.645
V3	0.499	0.009*	0.046	0.628	0.033	0.890
hMT+	0.324	0.025*	0.035	0.627	0.038	0.821
DRA	0.598	0.003*	0.074	0.472	0.060	0.779
VRA	0.806	< 0.001*	0.075	0.543	0.038	0.891

Asterisks indicate statistical significance at *p* < 0.05.
